# Supplementary material for: Glycation Leads to Increased Invasion of Glioblastoma Cells
Source: Cells. 2023 Apr 23;12(9):1219. doi: 10.3390/cells12091219 (PMC10177211; doi:10.3390/cells12091219)
Supplement: Supplementary file 1 [file cells-12-01219-s001.zip › cells-2264754-supplementary.pdf]

Supplementary Materials

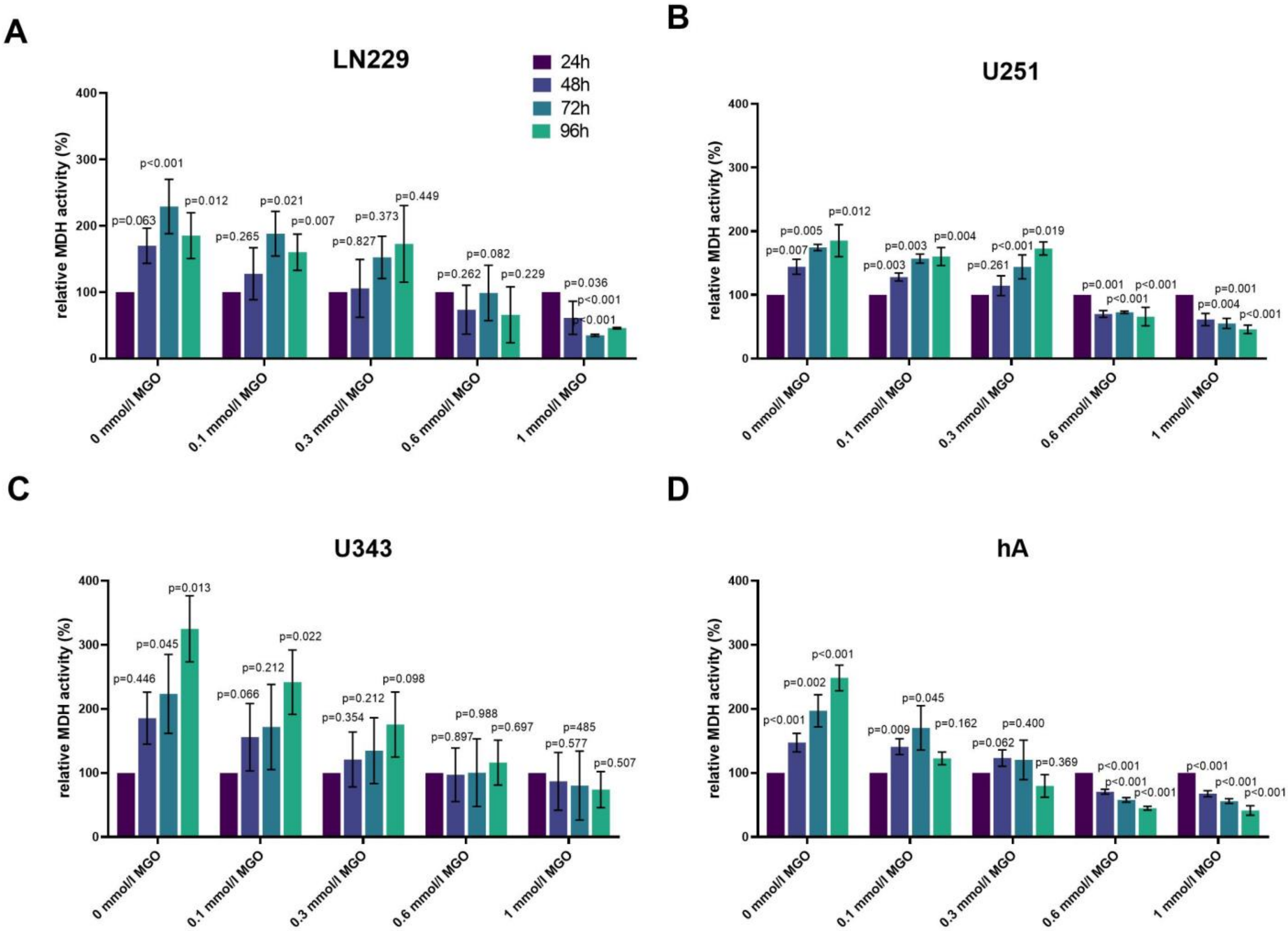

Figure S1. Cell vitality of glioma cell lines and hA after MGO treatment. The cell vitality of LN229 (**A**), U251 (**B**), U343 (**C**), and hA (**D**) cells was determined using an XTT assay after MGO treatment. Graphs show intracellular mitochondrial dehydrogenase (MDH) activity normalized to untreated cells after 24, 48, 72 and 96 hours. Student's t-test was performed for statistical analysis. Graphs represent the means and SD of three independent biological replicates.

Table S1. Quantification of microscope imaging of glioma cell lines and hA after 24 hours.

	0 mmol/L MGO	0.1 mmol/L MGO	0.3 mmol/l MGO	0.6 mmol/L MGO	1 mmol/L MGO
**LN229**	99.80%	99.89%	99.86%	99.74%	99.57%
**U251**	99.88%	94.65%	89.08%	91.27%	74.70%
**U343**	99.75%	99.35%	98.31%	91.67%	89.60%
**hA**	99.67%	98.68%	96.46%	35.29%	16.32%

The cell viability of LN229, U251, U343, and hA was determined using the IndentifyPrimaryObjects tool of the CellProfiler software (Version 4.2.4, Broad Institute, Massachusetts, USA).

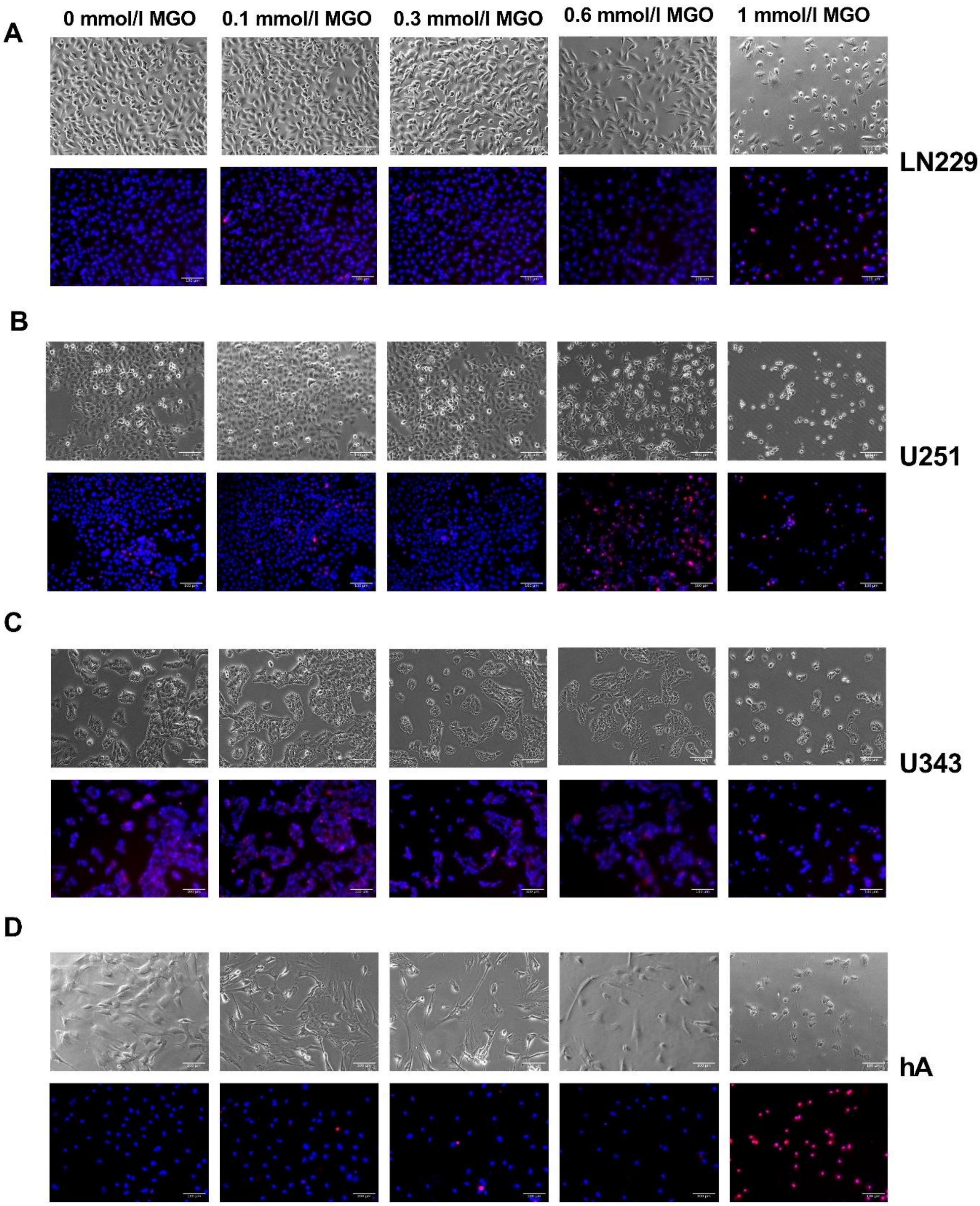

Figure S2. Microscope imaging of glioma cell lines and hA 48 hours after MGO treatment. Bright field (above) and fluorescence (below) microscope imaging of LN229 (A), U251 (B), U343 (C) and hA (D) 48hours after MGO treatment. Cells were stained with DAPI (blue) and propidium iodide (red). Scale bar = 100 μm.
